# Supplementary material for: Balance Training and Shooting Performance: The Role of Load and the Unstable Surface
Source: J Funct Morphol Kinesiol. 2024 Jan 3;9(1):17. doi: 10.3390/jfmk9010017 (PMC10801566; doi:10.3390/jfmk9010017)
Supplement: Supplementary file 1 [file jfmk-09-00017-s001.zip › jfmk-2795798-supplementary.pdf]

Table S1: The shooting parameters in control and experimental groups in the four conditions, before and after the 4-week balance training (onB: on balance; noL: no load; offB: off balance; L: with load).

	Before Training				After Training			
	onB-noL	onB-L	offB-noL	offB-L	onB-noL	onB-L	offB-noL	offB-L
				CONTROL GROUP				
Hold (sec)	3,00 ± 0,81	3,27 ± 1,00	2,46 ± 0,48	2,34 ± 0,32	3,52 ± 1,44	4,13 ± 1,37	2,74 ± 1,12	2,72 ± 1,15
Xdev (cm)	2,20 ± 0,57	2,58 ± 0,83	1,99 ± 0,45	3,08 ± 0,89	2,02 ± 0,42	2,34 ± 0,35	2,29 ± 0,52	2,99 ± 0,43
Ydev (cm)	2,16 ± 0,44	2,63 ± 0,71	2,16 ± 0,32	3,28 ± 0,47	1,94 ± 0,67	2,36 ± 0,65	2,35 ± 0,70	2,74 ± 0,64
Interval (sec)	5,82 ± 1,05	6,17 ± 1,30	4,93 ± 0,69	4,78 ± 0,38	6,61 ± 2,10	8,22 ± 1,32	6,52 ± 1,40	6,11 ± 1,16
RTV (AU)	1,03 ± 0,10	0,99 ± 0,11	1,01 ± 0,10	1,02 ± 0,11	1,02 ± 0,08	1,01 ± 0,14	0,99 ± 0,11	0,99 ± 0,10
				EXPERIMENTAL GROUP				
Hold (sec)	2,70 ± 1,94	2,30 ± 2,67	2,23 ± 1,22	1,94 ± 0,72	4,55 ± 1,29#	5,17 ± 1,30#*	4,08 ± 0,79#	4,47 ± 1,44#*
Xdev (cm)	2,00 ± 0,47	2,78 ± 0,72	1,97 ± 0,35	2,91 ± 0,49	1,54 ± 0,38#	2,00 ± 0,44#	1,35 ± 0,58#	2,29 ± 0,63#
Ydev (cm)	2,01 ± 0,21	2,45 ± 0,26	2,17 ± 0,37	2,98 ± 0,65	1,00 ± 0,27#	1,45 ± 0,18#	1,23 ± 0,35#	2,03 ± 0,59#
Interval (sec)	8,14 ± 2,71	9,67 ± 3,12	7,18 ± 2,44	7,90 ± 2,21	8,95 ± 2,94	9,38 ± 1,49	7,97 ± 2,12	9,44 ± 2,82
RTV (AU)	1,02 ± 0,10	1,07 ± 0,13	1,07 ± 0,14	1,00 ± 0,14	0,95 ± 0,04	0,97 ± 0,10	0,94 ± 0,12	0,93 ± 0,07

significant differences after the training program, $p < 0.01$; * significant differences compared to no load conditions, $p < 0.01$.
